# Inhibition of early steps in the lentiviral replication cycle by cathelicidin host defense peptides

**DOI:** 10.1186/1742-4690-2-2

**Published:** 2005-01-18

**Authors:** Lars Steinstraesser, Bettina Tippler, Janine Mertens, Evert Lamme, Heinz-Herbert Homann, Marcus Lehnhardt, Oliver Wildner, Hans-Ulrich Steinau, Klaus Überla

**Affiliations:** 1 Department for Plastic Surgery, BG University Hospital Bergmannsheil, Ruhr University Bochum, Buerkle-de-la- Camp Platz 1, 44789 Bochum, Germany; 2 Department of Molecular and Medical Virology, Ruhr University Bochum, Universitätsstraße 150, 44801 Bochum, Germany; 3 Department of Dermatology, University Medical Center Nijmegen, Geert Grooteplein 9, 6525 GA Nijmegen, Netherlands

## Abstract

**Background:**

The antibacterial activity of host defense peptides (HDP) is largely mediated by permeabilization of bacterial membranes. The lipid membrane of enveloped viruses might also be a target of antimicrobial peptides. Therefore, we screened a panel of naturally occurring HDPs representing different classes for inhibition of early, Env-independent steps in the HIV replication cycle. A lentiviral vector-based screening assay was used to determine the inhibitory effect of HDPs on early steps in the replication cycle and on cell metabolism.

**Results:**

Human LL37 and porcine Protegrin-1 specifically reduced lentiviral vector infectivity, whereas the reduction of luciferase activities observed at high concentrations of the other HDPs is primarily due to modulation of cellular activity and/ or cytotoxicity rather than antiviral activity. A retroviral vector was inhibited by LL37 and Protegrin-1 to similar extent, while no specific inhibition of adenoviral vector mediated gene transfer was observed. Specific inhibitory effects of Protegrin-1 were confirmed for wild type HIV-1.

**Conclusion:**

Although Protegrin-1 apparently inhibits an early step in the HIV-replication cycle, cytotoxic effects might limit its use as an antiviral agent unless the specificity for the virus can be improved.

## Background

As a barrier and immune organ, the gastrointestinal tract, lung and skin play a key role in protecting the body from a hostile environment [1]. The low incidence of infection at normal epithelial surfaces reflects the presence of innate, broad-spectrum antimicrobial defense mechanisms [2]. Host defense peptides (HDPs) of the innate immune response play an important role in the protective barrier function of the epithelia [3]. Host defense peptides have been isolated from diverse organisms, including plants, insects, bacteria and vertebrates [4]. Several classes of mammalian peptide antibiotics have been ascribed pivotal roles in innate immunity [5]. Among these are various cysteine-rich peptides such as defensins [6,7] and the more structurally diverse cathelicidins [8]. Produced as precursors, they require proteolytic processing to liberate the mature functional antimicrobial peptide. Cathelicidins contain a conserved N-terminal cathelin domain, and a structurally diverse C-terminal domain that possesses the peptide's antimicrobial activity. Rabbit CAP18 was the first cathelicidin precursor described, and its mature peptide has broad-spectrum antimicrobial activity [9]. Cathelicidins have since been identified in many other species including hCAP18/LL37 in humans [10], protegrins in swine [11-13], CRAMP in mice [14,15] and SMAP29 in sheep [16]. Many of these peptides demonstrate extremely broad-spectrum antimicrobial activity, including Gram positive and Gram negative bacteria and fungi [4,15]. In addition, they achieve bacterial killing much more rapidly than any commercially available antibiotic [17]. Recently, a new family of synthetic, α-helical HDPs called "ovispirins" was described [18-20]. Although some of these modified peptides had similar antimicrobial activity of naturally occurring peptides, they manifested appreciable cytotoxicity. We have demonstrated recently that variants of Ovispirin, the so called Novispirin peptides, displayed more favorable toxic/ therapeutic ratios *in vitro *and broad spectrum activity in infected rat burn model [21,22].

Some of these peptides are induced at epithelial surfaces in response to invading organisms [23-25]. Many HDPs kill microorganisms by causing membrane permeabilization, although not necessarily as their sole mode of action [26]. Some HDPs also direct chemotaxis, promote wound healing, angiogenesis and contribute to adaptive immunity by mobilizing memory T cells and immature dendritic cells [25,27]. Recent studies have also demonstrated antitumor activity after treatment with HDPs [28].

In addition, several antiviral activities were reported. Recently it has been demonstrated that rabbit neutrophil peptide alpha-defensin NP1 protects cells from infection with HSV-1 and 2 [29]. Other studies revealed that human neutrophil peptide HNP1 to 3 and Theta-defensins also inhibit HSV infection although by different mechanisms [30-32]. The ancestral human theta-defensins retrocyclin blocked HSV attachment [33]. The inhibition of adenovirus replication by the antimicrobial peptide awaits identification of a mechanism of action [30]. Anti-HIV activity of defensins were first reported 1993 by Nakashima and coworkers [34]. The alpha-defensins exhibited anti-HIV activity on at least two levels: directly inactivating virus particles; and affecting the ability of target CD4 cells to replicate the virus [35-37]. Binding to gp120 of HIV-1 and inhibition of HIV entry has also been identified as the mechanism of inhibition of HIV infection by theta defensins [38]. Due to their inhibitory effect on HIV-1 replication and due to an association of a single-nucleotide polymorphism in a beta defensin gene human beta-defensins might also play an important role in host defense against HIV-1 [39].

The antibacterial activity of HDPs is largely mediated by pore formation leading to permeablization of the bacterial membrane. Although some selectivity for bacterial membranes has been described, the lipid membrane of enveloped viruses might also be a target of antimicrobial peptides [32,40]. This might allow development of antiviral effector molecules for topical application against a broad spectrum of enveloped viruses. Targeting host cell-derived membrane components might be a particularly interesting approach to inhibit viruses that rapidly develop resistance to compounds directed against viral proteins such as HIV. Therefore, we screened a panel of different natural occurring and designer HDPs for Env-independent inhibition of HIV infection at an early step in the viral replication cycle.

## Results

**Figure 1:**
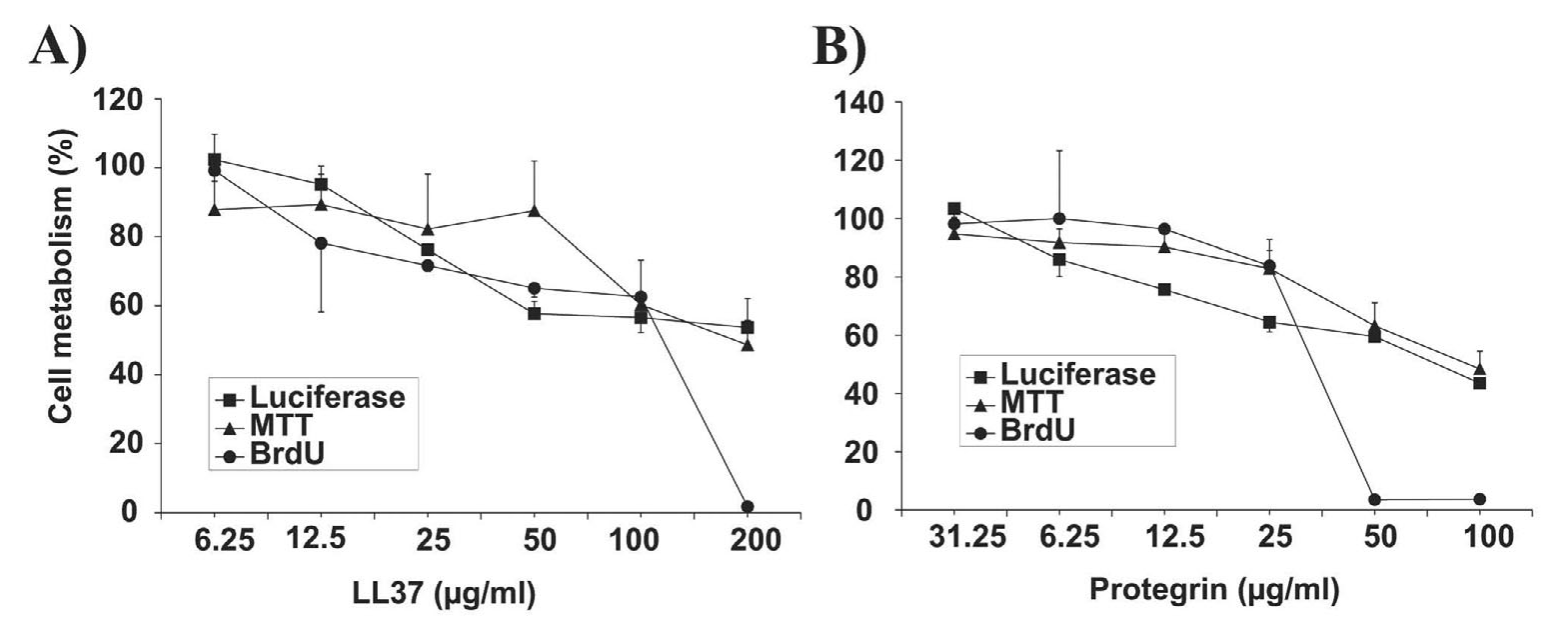
**Comparison of different cytotoxicity assays. **293A target cells stably transduced with the luciferase gene were incubated for 48 hours in the indicated concentrations of LL37 or Protegrin-1. Viability, cell proliferation and cell metabolism of parallel cultures were assessed by a standard MTT assay, Brd-U incorporation and the luciferase assay, respectively. Values are expressed as percentage of the values obtained from cultures without HDPs. The mean and the standard deviation of triplicates are given.

Given the potential cytotoxicity of HDPs, it was important to discriminate between modulation of cell metabolism and direct antiviral effects. We were concerned that HDPs could modulate host cell metabolism without affecting cell viability as assayed for example by standard MTT assays. We therefore decided to use immunodeficiency virus-based vectors transferring the luciferase gene to determine both, the HDP antiviral activity and modulation of cell metabolism. The luciferase activity of target cells, stably transduced with the lentiviral vector prior to HDP treatment should reveal any effect of HDPs on cellular transcriptional and translational efficacy. Treating cells with HDPs during infection with the lentiviral vector and comparison with results obtained with the stably transduced cells should then allow identifying HDPs that inhibit early steps in the viral replication cycle. To validate the luciferase-based assay for modulation of cell metabolism, 293 cells were stably transduced with a lentiviral vector transferring the luciferase gene. Incubation of transduced cells with increasing concentrations of Protegrin-1 and LL37 led to a dose-dependent inhibition of luciferase activity (Fig. 1). Comparison to the MTT test revealed a similar dose response curve, although the MTT test might be less sensitive at lower concentrations of the HDPs. Testing cell proliferation by a BrdU incorporation assay revealed a threshold level above which proliferation is strongly reduced. To allow side by side evaluation of cytotoxic and antiviral effects of HDPs with the same read-out the luciferase-based assay was used in most subsequent experiments.

A panel of HDPs containing members of the major classes of antimicrobial peptides were analyzed for inhibitory effects against lentiviral vectors. Given the variability of the viral envelope protein, we focused on identifying Env-independent inhibitory activities by using VSV-G pseudotyped lentiviral vectors. All antimicrobial peptides used in this study, human cathelicidin LL37, recombinant human β-Defensin-2, porcine Protegrin-1 (PG-1), fungal Plectasin and Novispirin G10, inhibited gram positive and gram negative bacteria revealing antimicrobial activity in the expected range (Table 1) and confirmed bioactivity of the peptides used.

**Table 1:** Summary of the radial diffusion assay results comparing host defense peptides with a clinically used antibiotic (Ampicillin)

	M E C (μg/ ml)					
	
Organisms	**Protegrin-1**	**LL–37**	**HBD 2**	**Plectasin**	**Novi- G10**	**Ampicillin**
*S. aureus*	0,91 ± 0,04	4,74 ± 0,1	9,13 ± 0,5	2,42 ± 0,4	4,2 ± 0,4	10,50 ± 0,2
*S. epidermidis*	4,48 ± 0,2	13,09 ± 0,6	8,96 ± 0,2	8,20 ± 0,5	2,8 ± 0,02	ND
*E. faecalis*	4,46 ± 0,3	13,61 ± 0,7	-	10,60 ± 0,7	4,3 ± 0,3	28,85 ± 0,6

*P. aeruginosa*	3,3 ± 1,1	12, 28 ± 0,5	6,87 ± 0,8	> 128	1,56 ± 0,4	ND
*E. coli*	2 ± 0,1	11,66 ± 1,5	3,66 ± 0,3	79,10 ± 0,3	1,63 ± 0,3	18,9 ± 0,9
*A. baumanii*	5,01 ± 0,2	13,13 ± 0,2	3,19 ± 0,5	> 128	4,5 ± 0,02	ND

All clinical isolates from human wounds showed significant sensitivity to HDPs. ND: not determined. MEC: minimal effectory concentration. MEC > 128 means that the tested bacteria is not susceptible to the drug tested. Depicted data consists of 3 individual experiments; each condition was performed in quadruplicates

**Figure 2:**
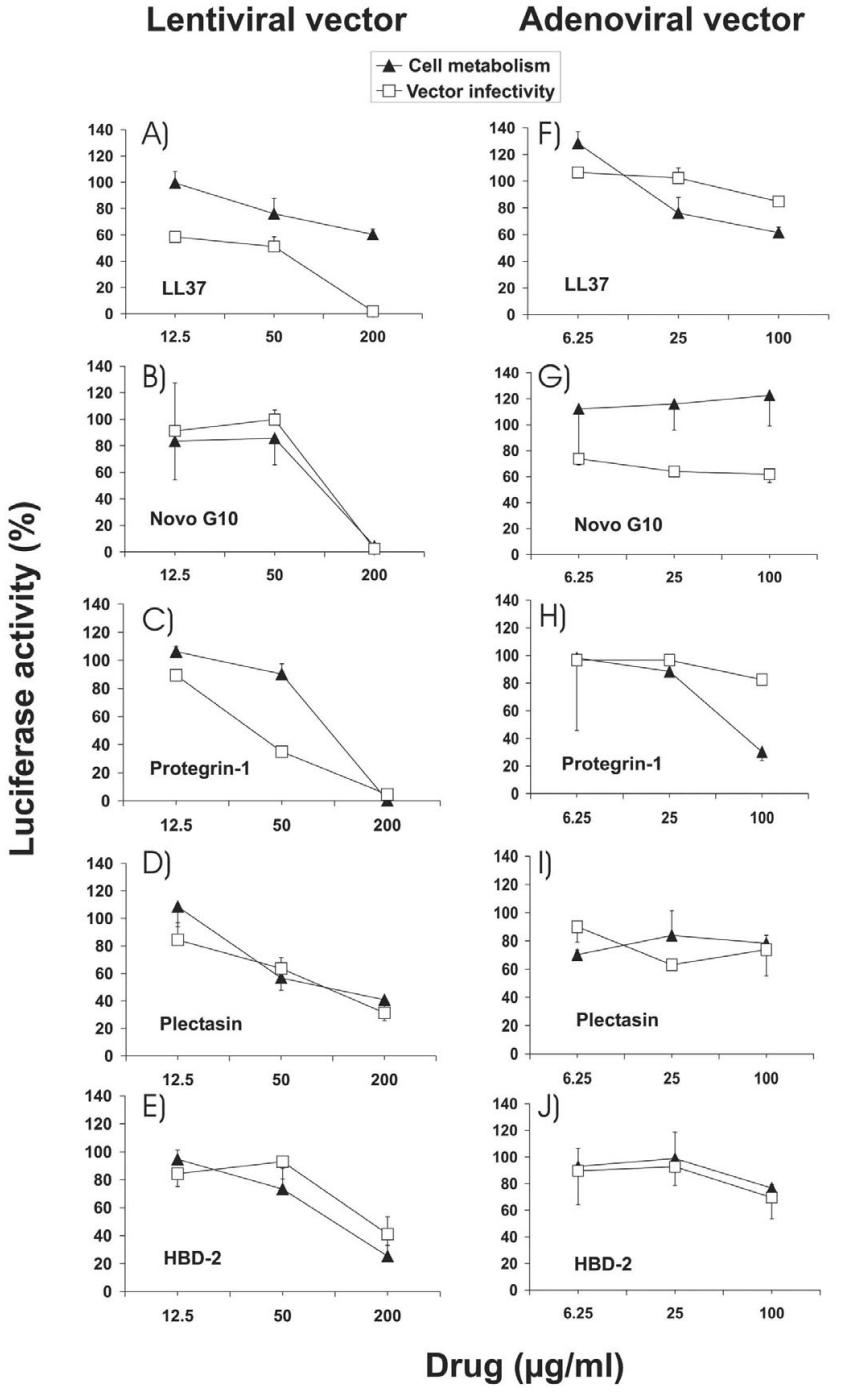
**Inhibitory activity of HDPs against lentiviral and adenoviral vectors. **Percent luciferase activity of 293A target cells transduced in the presence of the indicated amounts of HDP with the VLΔBH lentiviral vector (A to E) or an adenoviral vector (F to J) both transferring the luciferase gene is shown. Modulation of cell metabolism was investigated in parallel by incubating 293A target cells stably transduced with a luciferase gene with the indicated amounts of HDP. The luciferase activity is expressed as percentage of the luciferase activity of cells cultured in the absence of HDP. The mean and the standard deviation of triplicates are given.

**Figure 3:**
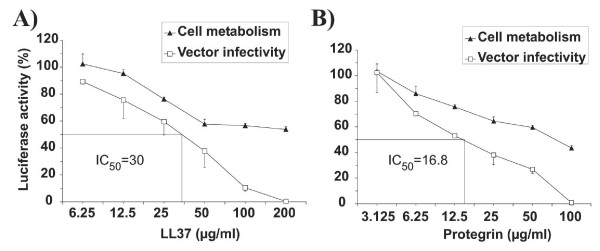
**50% inhibitory concentrations of LL37 and Protegrin-1. **Two-fold serial dilutions were used to determine the IC_50_s of LL37 (A) and Protegrin-1 for the VLΔBH vector (B). Modulation of cell metabolism was investigated in parallel by incubating 293A target cells stably transduced with a luciferase gene with the indicated amounts of HDP. The luciferase activity is expressed as percentage of the luciferase activity of cells cultured in the absence of HDP. The mean and the standard deviation of triplicates are given.

The inhibitory effect against the lentiviral vector was determined by preincubation of the vector with human cathelicidin LL37, recombinant human β-Defensin-2, porcine PG-1, fungal Plectasin and Novispirin G10 for 30 minutes in increasing concentrations of peptide prior to the addition of vectors with antimicrobial peptide to the target cells. Stably transduced target cells were incubated in parallel with the HDPs to detect effects on cell metabolism. Luciferase activities were determined 2 days after infection. All HDPs led to a reduction in luciferase activity of cells transduced with the lentiviral vector (Fig. 2A–E), but most of them also reduced the luciferase activity of stably transduced target cells. Specific inhibition of early steps of infection was only seen for the cathelicidin LL37 and PG-1. As an additional control for the specificity of inhibition, a non-enveloped adenoviral vector transferring the luciferase gene was also incubated with the same panel of HDPs. None of the HDPs led to a dose-dependent inhibition of early steps of the adenoviral replication cycle (Fig. 2F–J). Two-fold serial dilutions were used to determine 50% inhibitory concentrations (IC_50_) of LL37 and PG-1, resulting in IC_50_s of approximately 30 μg/ml and 16.8 μg/ml, respectively (Fig. 3).

**Figure 4:**
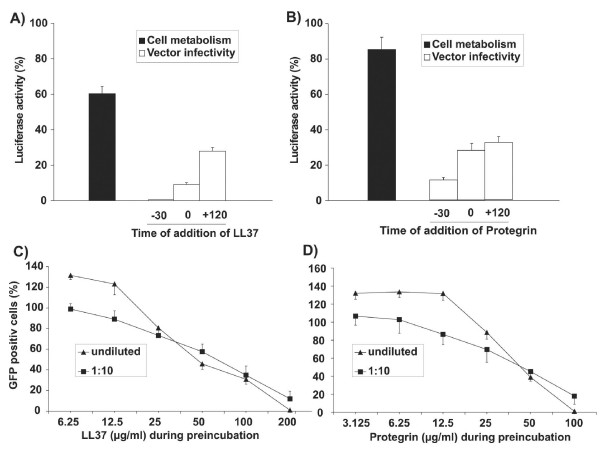
**Time and concentration dependent inhibition of lentiviral vectors by LL37 and Protegrin-1. **The lentiviral vector VLΔBH transferring the luciferase gene was either preincubated with 200 μg/ml of LL37 (A) or 50 μg/ml of Protegrin-1 (B) for 30 minutes (-30) or added simultaneously (0) with LL37 and Protegrin-1 to 293A target cells. Target cells were also preincubated for 120 minutes with the lentiviral vector prior to addition of LL37 and Protegrin-1. Two days after infection luciferase activities were determined as percentage of luciferase activities of cells cultured in the absence of HDPs. Cells stably transduced with the luciferase gene were also cultured in the presence and absence of LL37 and PG-1 to determine the effect of HDPs on the cell metabolism. The mean and the standard deviation of triplicates is given. A lentiviral vector transferring the GFP gene (HIV-CSCG) was incubated for 30 minutes at the indicated concentrations of LL37 (C) or Protegrin-1 (D). The vector was then added directly to 293A target cells (undiluted) or after a 1:10 dilution in medium lacking the HDPs. The number of GFP positive cells at each HDP concentration is given as percentage of GFP-positive cells of cultures transduced with diluted and undiluted vectors in the absence of HDPs. The mean and standard deviation of triplicates are shown.

In initial attempts to characterize the mechanism of inhibition of lentiviral vector infectivity, LL37 and PG-1 were added at different time points during infection: both HDPs were either preincubated with the vector preparation for 30 minutes prior to addition to the cells or the HDPs and the vector were added simultaneously to the cells (Fig. 4A+B). In addition, cells were first infected with the lentiviral vector for two hours prior to addition of the HDPs. LL37 and PG-1 exerted the strongest inhibition after preincubation of HDPs and the lentiviral vectors, indicating a direct effect on infectivity of the vector particles. However, adding LL37 and PG-1 two hours after incubation of cells with the lentiviral vector also led to a stronger reduction of luciferase activity than observed after incubation of cells stably transduced with the luciferase gene suggesting a second target in the infection cycle that is affected by LL37 and PG-1. To further discriminate between direct inhibitory effects on vector particles and effects mediated by potential HDP cell interactions lentiviral vector particles were first incubated with LL37 and PG-1 for 30 minutes and then added either undiluted or at a 1:10 dilution to the target cells. Due to a 10-fold lower HDP concentration in the latter cultures, vector infectivity should be reduced to a lesser extent, if inhibitory effects are mediated by cellular targets. Comparable dose-dependent inhibition curves (Fig 4C,D) of diluted and undiluted vectors demonstrate that the inhibitory effects of LL37 and PG-1 depend on the HDP concentration during preincubation of the vector particles and not on the HDP concentration during subsequent cell culture.

**Figure 5:**
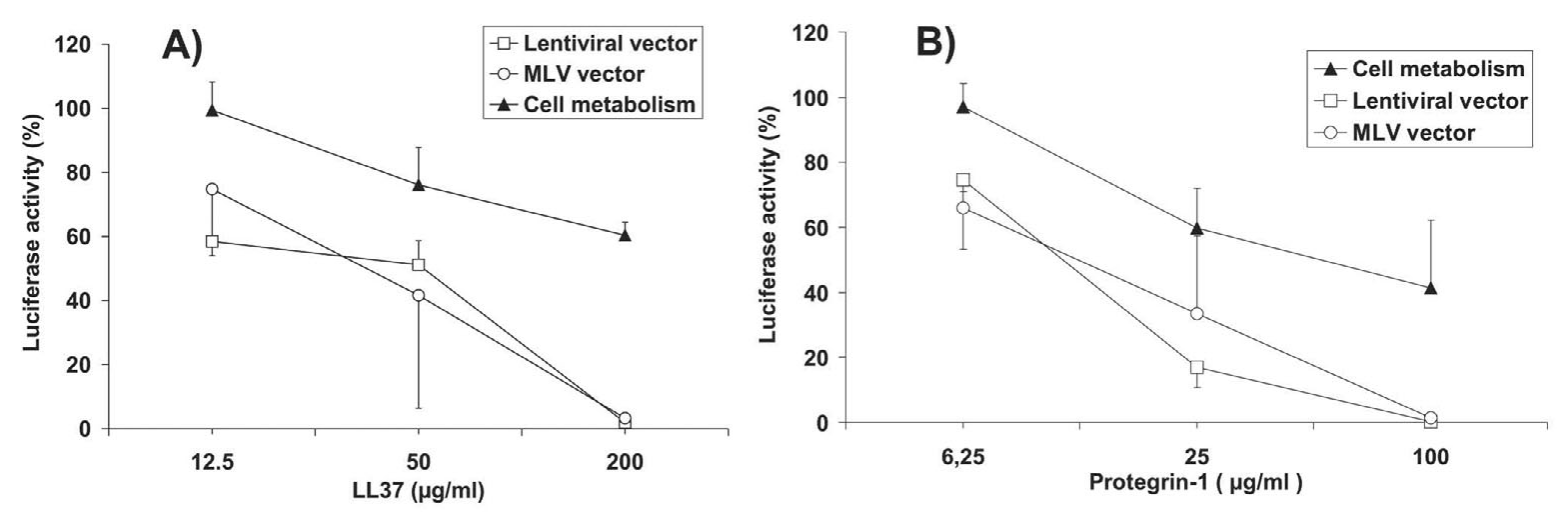
**Comparative analysis of inhibition of lentiviral and retroviral vector infectivity. **Percent luciferase activity of 293A target cells transduced in the presence of the indicated amounts of HDP with a lentiviral vector (VLΔBH) or a retroviral vector (pRV-172) both transferring the luciferase gene is shown. Modulation of cell metabolism was investigated in parallel by incubating 293A target cells stably transduced with a luciferase gene with the indicated amounts of HDP. The luciferase activity is expressed as percentage of the luciferase activity of cells cultured in the absence of HDP. The mean and the standard deviation of triplicates of two independent experiments are shown.

The lentiviral vector used in this study had been pseudotyped with the G protein of vesicular stomatitis virus (VSV-G). To evaluate whether LL37 and PG-1 directly target VSV-G or a lentiviral protein, the inhibitory effect of these HDP against the lentiviral vector was compared side by side to their effect on a retroviral vector containing the amphotropic MLV Env for entry into target cells. The dose dependent reduction in the luciferase activity in the target cells was very similar in cells transduced with the lentiviral or the MLV vector (Fig. 5).

**Figure 6:**
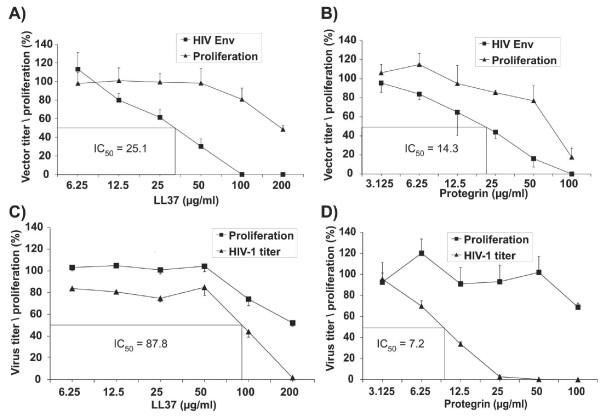
**Inhibitory effects of Protegrin-1 and LL37 on HIV-1 Env mediated vector entry (A, B) and HIV-1 infection (C,D). **A lentiviral vector transferring the GFP gene (VGΔBH-SIN) was incubated at increasing concentrations of LL37 (A) or Protegrin-1 (B) prior to transduction of P4CCR5 cells. The vector titer is given as percentage of the titer of the vector incubated in the absence of HDPs. Wild type HIV-1 was incubated with increasing concentrations of LL37 (C) and Protegrin-1 (D). The virus titer was subsequently determined on P4CCR5 indicator cells and is expressed as percentage of the titer of the untreated HIV-1 virus stock. The toxicity of LL37 and Protegrin-1 was determined in parallel using the BrdU incorporation assay.

The inhibition of HIV vectors containing the HIV-1 envelope by LL37 and PG-1 were studied on P4CCR5 cells expressing CD4 and coreceptors. IC_50_s of 25 μg/ml and 14 μg/ml were observed for LL37 and PG-1, respectively (Fig 6A,B), while only minimal inhibitory effects on cell proliferation were detected at these concentrations. Inhibition of early steps of wild type HIV-1 infection by LL37 and PG-1 was also evaluated on P4CCR5 indicator cells, which produce beta-Galactosidase upon expression of the viral tat gene after infection. The BrdU incorporation assay was used to evaluate modulation of cell function. A dose dependent reduction of the titer of HIV-1 on P4CCR5 cells was observed for both HDPs. However, the IC_50 _of LL37 was approximately 3-fold higher than the IC_50 _previously determined for the lentiviral vectors resulting in a narrow gap between antiviral and antiproliferative effects of LL37. In contrast, the IC_50 _of PG-1 was below 10 μg/ml, while inhibitory effects on cell proliferation were not observed up to concentrations of 50 μg/ml.

## Discussion

From the panel of five HDP studied, the cathelicidin LL37 and PG-1 were found to specifically inhibit lentiviral and retroviral vector, but not adenoviral vector infectivity. The strongest inhibition was seen if the lentiviral vectors were preincubated with LL37 and PG-1. This suggests that these HDPs directly interacted with the vector particles, which is consistent with our observation that inhibition was dependent on the HDP concentration during preincubation of the vectors with HDPs, but not on the HDP concentration during infection of the cells. Since lentiviral vectors and retroviral vectors were inhibited to a similar degree although they do not share any viral protein, the target for the HDP on the particles is probably cell-derived. This could either be the lipid membrane derived from the cell, which surrounds the vector particles or cellular membrane proteins that are frequently incorporated in lentiviral and retroviral particles during budding [41]. A permeabilizing effect of LL37 and PG-1 on the viral particles would be consistent with our data, but other mechanisms of inhibition cannot be excluded. While the inhibitory effect of PG-1 was also detected with wild type HIV-1 on P4CCR5 cells, LL37 inhibited HIV-1 to lesser degree then the lentiviral vectors. Due an IC_50 _of 88 μg/ml against wild type HIV-1, it is questionable whether LL37 concentrations are sufficiently high at mucosal membranes to play a role in host defense against HIV-1.

## Conclusions

Modulation of cell metabolism was generally seen at concentrations of HDPs exceeding 50 μg/ml, while the MEC of the antibacterial activity ranged from 1 to 10 μg/ml. This might leave a sufficient window for therapeutic intervention of bacterial infection. However, for the treatment of HIV-1, the therapeutic window of LL37 and PG-1 is rather narrow. It should also be noted that the HDP-induced modulation of cell metabolism and cytotoxicity can be cell type dependent. Therefore, increasing the selectivity of HDPs for early steps in the viral replication cycle seems to be necessary for further development of the human cathelicidin LL37 and the porcine Protegrin-1 as antiviral agents for systemic or topical applications.

## Methods

### Preparation of vectors transferring the luciferase or GFP genes

To generate lentiviral vector particles transferring the luciferase gene, a codon-optimized (Geneart GmbH, Regensburg, Germany) HIV-1 *gag-pol *expression plasmid (Hgp^syn^) [42] and a VSV-G expression plasmid (pHIT-G) [43] were used to package the SIV-based vector VLΔBH. This vector contains the luciferase gene replacing the GFP gene of VGΔBH [44].5 μg of Hgp^syn^, 2 μg of pHIT-G and 5 μg of VLΔBH were transiently cotransfected by the CaPO_4_coprecipitation method into 293T cells as previously described [45]. An HIV vector construct containing the GFP reporter gene (HIV-CSCG) [46] was also used to prepare lentiviral vector particles by cotransfection with Hgp^syn^, pcTat [47], pcRev [47] and pHIT-G or pSVIIIenv3-2, an HIV-1 envelope expression plasmid [48]. The MLV vectors were prepared by cotransfection of pHIT-456, pHIT-60 and pRV-172 [49]. Two days after transfection, the supernatants were cleared from cellular debris by low speed centrifugation (10 minutes, 1000 × g) and filtration through 0.2 μm filters from Roth (Karlsruhe, Germany). Aliquots were stored at -80°C.

### Construction and Production of Ad.OW126 Vector

Beginning with a first generation E1- and E3-deleted adenoviral vector, we generated a replication-competent adenoviral vector Ad.OW126, which harbors in the E1 region the firefly luciferase cDNA (subcloned from pGEM-Luc (Promega, Madison, WI)), a IRES element [50,51], and an Ad5 E1A ΔE1B-55K gene. The entire expression cassette is driven by the human CMV-IE promoter in parallel to the transcriptional orientation of the adenovirus E1 gene products and terminated by the bovine growth hormone polyadenylation site. The expression cassette was flanked upstream by the Ad5 packaging sequence and downstream by the Ad5 pIX. The Ad.OW126 vector was generated by *in vitro *ligation [52] to H5*dl*327 (kindly provided by T. Shenk, Princeton University, Princeton, NJ), utilizing the unique *Bst*1107 I restriction site. The vector was propagated in 293 cells and purified by two rounds of CsCl density centrifugation [53], dialyzed (Slide-A-Lyzer, Pierce, Rockford, IL) against 1500 ml of PBS with 1 mM MgCl_2 _and 10% glycerol four-times (1 hour each) at 4°C, and stored at -80°C until use. The concentration of the vector was determined by measuring absorbency at 260 nm [54], and the infectious titer was determined by plaque assay on 293 cells [55]. The ratio of infectious to non-infectious virus particles was approximately 1:80.

### Generation of 293A cells stably transduced with a luciferase gene

To stably transduce the luciferase gene into 293A cells, a self-inactivating version of VLΔBH, VLΔBH-SIN similar to VGΔBH-SIN [44] was packaged by cotransfection with Hgp^syn ^and pHIT-G. 293A cells were plated in 24 well plates at a density of 50.000 cells / well and transduced with 200 μl of VLΔBH-SIN vector for two hours. Two days after plating cells were transferred to one well of a six well plate and transduced again with 1 ml of VLΔBH-SIN vector. Cells were subsequently expanded resulting in 293-Luc cells.

### Luciferase assay

The supernatant of infected 293A or 293-Luc cells, cultured in 96 well plates was removed and cells were lysed in 50 μl of cell lysis buffer (Promega, Pittsburgh, PA). 20 μl of the cell lysates were used in the firefly luciferase assay system of Promega as described by the manufacturer. Each single value of the triplicates was expressed as percent of the mean of triplicates of control cultures infected with the same vector in the absence of HDPs and the mean and the standard deviation of the percent values was calculated for each triplicate.

### Host defense peptides

The antimicrobial peptides (human LL37, porcine PG1-1, mutants from the ovine SAP29: Novispirin G10 and fungal Plectasin) used in this study were prepared by solid phase synthesis and purified by RP-HPLC. Recombinant human β-Defensin-2 was produced by a molecular farming approach in transgenic potato tubers and purified by perfusion chromatography (data not shown). The peptides (≥ 98% pure) were dissolved in 0.01% acetic acid and used for all *in vitro *and *in vivo *studies. Potential endotoxin contamination was monitored with the chromogenic *Limulus *amoebocyte lysate assay (BioWhittaker, Walkersville, MD) using *Escherichia coli *endotoxin (supplied with the kit) as the standard. Endotoxin levels for the peptides were not detectable.

### Bacteria

The following strains were used in this study: Gram-negative strains: *Acinetobacter baumannii *(ATCC 19606), *Escherichia coli *(ATCC 25922) and *Pseudomonas aeruginosa *(ATCC 27853) Gram-positive strains: *Staphylococcus aureus *(ATCC 25923), *Staphylococcus epidermidis *(ATCC 12228) and *Enterococcus faecalis *(ATCC 29212).

All bacterial strains were analyzed with API test strips (BioMerieux, Hazelwood, MO) to confirm identity and aliquots were stored frozen in 50 % skim milk at -80°C. Bacteria were grown overnight in trypticase soy broth (Becton Dickinson, Franklin Lakes, NJ) at 275 rpm and 37°C. An aliquot of the resulting stationary phase cultures was then transferred to 20 ml of trypticase soy broth and incubated at 37°C for 2.5 hours to reach log phase. This subculture was transferred to a 50 ml conical polystyrene tube and centrifuged for 10 min at 4°C at 880 g. The bacterial pellet was washed once with chilled phosphate buffered saline, pH 7.4, and resuspended in 5 ml of the same cold buffer. One milliliter was removed to measure its optical density at 620 nm. The bacterial concentration was calculated from the following formula: CFU/ml = OD_620 _× 2.5 × 10^8^.

### Growth Inhibition Assay

To monitor bacterial growth inhibition *in vitro *a radial diffusion assay was performed as previously described [56]. Briefly, the underlay agar consisted of 1% agarose (A-6013, Sigma Chemical, St. Louis, MO) and 0.3 mg/ml trypticase soy broth (TSB) powder in 10 mM sodium phosphate with 100 mM NaCl (normal salt medium), pH 7.4. Bacteria (approximately 5 × 10^6 ^CFU) were mixed with 10 ml of underlay gel (43°C) and immediately poured into square 9 × 9 cm petri dishes. A series of 3 mm wells was punched after the agarose solidified. After appropriate serial dilutions were done, 5 μl of HDP, vancomycin (Abbott Labs, Chicago, IL), gentamicin, ciprofloxacin, or fluconazole (Sigma-Aldrich, St. Louis, MO) were added to the designated wells. Plates were incubated at 37°C for 3 hours. The bacteria-containing layer was covered with a 10 ml overlay of the nutrient rich agar. The overlay agar consisted of 6% (w/v) TSB and 1% agarose in PBS for all assays. After 18 h of incubation at 37°C, the plates were stained with 0.001% Coomassie blue for 10 h. The clear zones (bacterial growth inhibition) around the punched wells indicated antibacterial activity. The diameters of the clear zones were converted into units by subtracting the well diameter and multiplying the difference by 10. Results were plotted using a semi log scale and correlation coefficients and X-intercepts obtained from linear regression analysis. The minimal effective concentration (MEC) corresponded to the X-intercept value. All assays were performed in triplicates and repeated at least once.

### Cytotoxicity and proliferation Assay

293-Luc cells were plated in 96 well plates at a density of 2 × 10^3 ^cells / well. After 48 hours, 50 μl of MTT-solution (3 mg/ml) was added and incubated at 37° C under 5% CO_2 _for 1 hour. After this time medium was removed and 100 μl 0.04 N HCL + 10% SDS was used to dissolve the resulting blue formazan crystals in living cells. The optical density was determined at 550 nm. Each single value of the triplicates was expressed as percent of the mean of triplicates of control cultures infected with the same vector in the absence of HDPs and the mean and the standard deviation of the percent values was calculated for each triplicate. In addition the BrdU Cell proliferation ELISA with chemiluminescence detection (Roche Diagnostics GmbH, Mannheim, Germany) was performed. After 293-Luc cells or P4CCR5 cells were plated in 96 well plates at a density of 2 × 10^3 ^cells/well BrdU (5-bromo-2'-deoxyuridine) was added to the cells with a resulting concentration of 10 μM for the last 22 h of the incubation period. After removing the culture medium, the cells were fixed and DNA was denatured in one step with Fixdenat. Thereafter the cells were incubated with Anti-BrdU-POD for 1 h at room temperature. The chemiluminescence detection was measured after automatic injection of substrate solution with a microplate-luminometer (Orion, Berthold detection systems, Pforzheim, Germany).

### Inhibition of vector infectivity

To determine the effect of HDPs on vector infectivity and cell metabolism, 293A target cells were plated in triplicates into 96-well plates at 2 × 10^3 ^cells / well. After overnight incubation, the supernatant of the wells were removed and replaced by 25 μl vector preparation and 25 μl HDP at twice the final concentration indicated. 50 μl fresh medium with HDP at the final concentration indicated was added after two hours. One (adenoviral vector) or two (lentiviral and retroviral vector) days after infection, the supernatant was removed and 50 μl of cell lysis buffer (Promega) was added. Lysates were stored at -80°C until determination of the luciferase activity of the extracts. The affect of HDPs on cell metabolism was determined in parallel by plating 293-Luc cells exactly the same way as 293A cells and the luciferase activity was determined after one (as a control to inhibition of adenoviral vector infectivity) or two (as a control of lentiviral infectivity) days of incubation with HDPs. For GFP-expressing vectors, vector titers were calculated from the number of GFP positive cells per well as previously described [45] and the BrdU incorporation assay was used to monitor cytotoxic effects.

### Inhibition of HIV-1

Stocks of HIV-1 were generated by transient transfection of 293T cells with the molecular clone pNL4-3. 25 μl of the virus stock were incubated for 30 minutes at room temperature with 25 μl of LL37 or PG-1 adjusted to twice the final concentration indicated. The mixture was added to P4CCR5 cells plated the day before at a density of 2 × 10^3 ^cells / well of 96 well plate. 50 μl fresh medium with HDP at the final concentration indicated was added after two hours. Two days after infection, the supernatant was removed and cells were stained by X-Gal. The number of infected cells per well were counted in the microscope.

## Competing interest

The author(s) declare that they have no competing interests.

## Authors' contributions

LS, BT and JM performed most of the experiments. LS, OW, ML, EL, HH, HS and KU participated in the experimental design, data interpretation and writing of the manuscript.
